# Teleworking to Support Accommodation, Inclusion, and Health of Aging Workers: Protocol for a Study to Design, Validate, and Test a Reflective Application Guide

**DOI:** 10.2196/46114

**Published:** 2023-05-25

**Authors:** Alexandra Lecours, Quan Nha Hong, Joanie Maclure, Roxanne Bédard-Mercier, Claude Vincent, Normand Boucher, Marie-Michèle Lord

**Affiliations:** 1 Département d'ergothérapie Université du Québec à Trois-Rivières Trois-Rivières, QC Canada; 2 Centre interdisciplinaire de recherche en réadaptation et intégration sociale Québec, QC Canada; 3 École de réadaptation, Université de Montréal Montréal, QC Canada; 4 Département de réadaptation, Université Laval Québec, QC Canada

**Keywords:** aging workers, health, intervention, qualitative research, telecommute, telework

## Abstract

**Background:**

Aging workers constitute a growing population in many countries and form an indispensable and qualified resource, especially in the context of the labor shortage. Despite work’s many benefits for individuals, organizations, and societies, it also presents several risks and challenges that may lead to occupational injuries. Thus, rehabilitation professionals and managers working with this emerging and unique clientele during their return to work after an absence often lack the tools and skills to support them, especially in the changing world of work that includes the rise of telework. Indeed, as an increasingly present work arrangement, telework has the potential to be used as an accommodation modality that can facilitate inclusion and healthy participation in the workplace. However, the implications of this topic for aging workers require study.

**Objective:**

This paper presents the protocol of a study that aims to develop a reflective telework application guide to support the accommodation, inclusion, and health of aging workers after an absence from work. Specifically, this study will (1) explore the experience of aging workers, managers, and rehabilitation professionals regarding telework and its impact on accommodation, inclusion, and health; (2) use a validated logic model to design a reflective application guide; and (3) test and evaluate the guide.

**Methods:**

Following a 3-phase developmental research design, individual interviews with aging teleworkers, managers, and rehabilitation professionals will enable the collection of qualitative data to be used in generating a logic model of levers and good practices, leading to the creation of a reflective application guide. Validation of this guide by workers and managers to measure its acceptability and applicability in daily life will precede its implementation.

**Results:**

Data collection began in spring 2023 and initial results are expected in fall 2023. This study aims to generate a concrete tool—namely, the reflective telework application guide—that rehabilitation professionals could use to support managers and aging workers during their return to work through the healthy use of telework. All phases of the study include conducting dissemination activities to share the results of the project and increase its sustainability potential (ie, publication through social networks, podcasts, conferences, and scientific publications).

**Conclusions:**

As the first of its kind, this project aims to produce innovative impacts at several levels, including practical, scientific, and societal impacts. In addition, the results will provide healthy solutions to the labor shortage in a changing world of work, where digital and teleworking are becoming increasingly important.

**International Registered Report Identifier (IRRID):**

DERR1-10.2196/46114

## Introduction

The number of aging workers (ie, aged 55 years or over [1]) is growing worldwide and forming an indispensable population contributing to economic life, especially in the context of a critical labor shortage. In Canada, this workforce represents more than one-third of the working-age population, almost 40% of whom are active in the labor market [2]. These are the highest proportions that the statistical account of work in Canada has noted. In the United States, the only group of workers on the rise since the mid-1990s are workers over the age of 55 years [3]. Moreover, they represent the group of workers that will grow the most by 2030 [4]. The European Union is also experiencing a similar situation, with a 17% increase in workers aged 55 to 64 years since 2009 [5].

This increase raises awareness of the importance of this group of workers who have specific characteristics differentiating them from other active generations at work. Indeed, this older generation of workers has distinctive values regarding work. For instance, older workers seem less likely than other generations to value the extrinsic advantages of work less than such intrinsic advantages as social contacts, challenges, and opportunities for responsibility [6]. In addition, some authors report that the baby boomer generation (ie, people born between 1946 and 1964 [7]) tends to show the greatest employer loyalty and a lower propensity than younger workers to leave their jobs [8]. These workers are also subject to particular representations by their younger colleagues. The results of a study on stereotypes of older workers show that others generally perceive them as more faithful but less likely to be fast adapters to technology [9].

Although work leads to many benefits for aging workers, such as developing a sense of belonging or ensuring financial security, it also carries risks [10]. Aging workers face higher probabilities of experiencing one or more periods of disability, either due to an occupational injury or a personal medical reason [10]. Moreover, the severity of the injury and the duration of disabling conditions escalate with age [11,12]. Indeed, workers aged 55 years and over are twice as likely as their younger colleagues to have a work-related accident and 6 times more likely than those colleagues to develop a musculoskeletal disorder [11]. Some authors report that gaps between workers’ abilities and job demands [13] as well as the decline of certain senses (eg, vision and audition [14]) partly explain these differences. Moreover, a major difference of nearly 100 days exists between the average duration of the disability period following an occupational injury for workers at least 55 years of age and that of younger workers aged 15 to 24 years [15]. Factors related to injury severity, presence of comorbidities, and tissue repair speed may mean longer periods of disability for aging workers [12,16], contributing to a marked increase in their use of rehabilitation services [17].

The increasing rehabilitation needs of aging workers create changes in the profile of the rehabilitation-service clientele [18]. Rehabilitation professionals now work with this emerging population and its distinctive characteristics. Professionals are generally aware of the growing presence of aging workers among the clients receiving their services. Nonetheless, they often do not know how to respond to this singular population in a changing world of work, and most clinical settings have not yet added specific services to meet those needs [18]. In addition, managers need resources to support aging workers in their return to work after an absence. Indeed, a recent scoping review revealed a gap between the needs of aging workers returning to work and the responses from managers [19]. Therefore, developing resources to help rehabilitation professionals support and guide managers and aging workers is essential, considering their singular characteristics.

The situation is even more complex because the COVID-19 pandemic fundamentally changed the way many people do their work, forcing a shift to digital work and telework [20,21]. As we emerge from this period of crisis, telework is clearly a work arrangement that will remain [22], with its advantages and challenges. Indeed, the research on telework during the pandemic has exploded, revealing some health benefits for individuals (eg, schedule flexibility [23]) but also challenges (eg, disconnection difficulties [24]) that may specifically impact aging workers’ mental health [25]. Other authors report the added value of telework in promoting inclusion and health for all worker populations [26,27], especially such marginalized populations as aging workers. While telework had been an exceptional accommodation for some workers (eg, workers with physical disabilities) from the 1990s until the late 2010s [28,29], workplace stakeholders more widely accept and recognize it now, resulting in less marginalization of workers who benefit from it.

Studies have highlighted the potential of telework as an accommodation for people living with specific health conditions as they feel more able to perform their work while managing their fatigue or pain levels [27,30-32]. Thus, using telework to accommodate individuals who may have challenges performing work in a conventional manner after a period of absence may be of interest. Indeed, this form of work provides “invisibility,” ensuring greater equality [26,27] as well as protection against prejudice and discrimination [31,33]. Some exploratory work suggests ways to use telework as a modality to facilitate accommodation, inclusion, and health for unique worker populations, such as people with physical disabilities [20,21] or neurodivergent workers [22]. Nonetheless, to our knowledge, there are not yet guidelines for ensuring a healthy application of telework for aging people. Recent research results have suggested that telework is particularly challenging for aging workers [34]. How can we ensure that this work arrangement promotes the accommodation, inclusion, and health of this workforce?

To optimize the contribution of all available people and reduce the labor shortage while promoting the principles of equity, diversity, and inclusion in the workplace, addressing this contemporary issue is important. Developing innovative tools to guide the healthy use of telework among aging people who have experienced an absence and improving knowledge about the use of telework as an accommodation modality for aging workers could help equip the rehabilitation professionals who support managers and workers in the trajectory of rehabilitation, return, and stay at work.

This study aims to develop a reflective telework application guide to support the accommodation, inclusion, and health of aging workers after an absence. Specifically, this study (1) explores the telework experience of aging workers, managers, and rehabilitation professionals; (2) designs the reflective application guide on the basis of a validated logic model; and (3) tests and evaluates the guide.

## Methods

### Design

**Figure 1:**
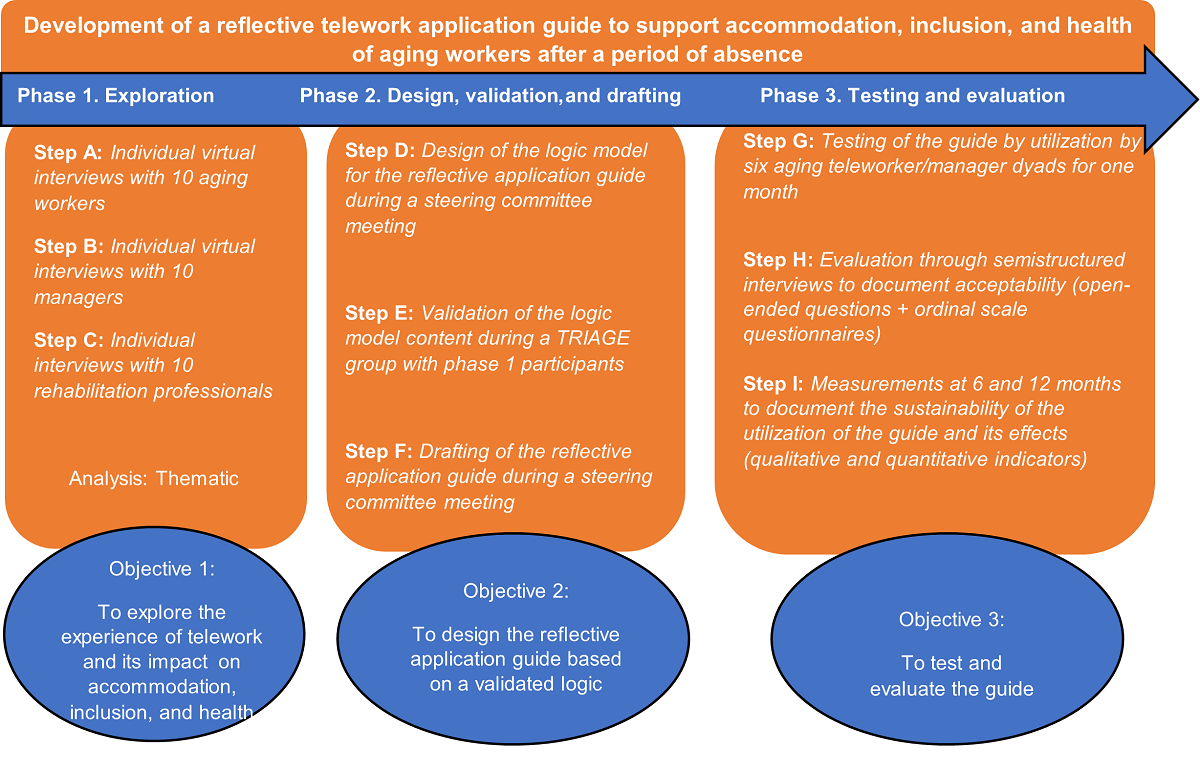
Phases, steps, and objectives of the research project. TRIAGE: technique for research of information by animation of a group of experts.

This project will proceed according to a developmental research design [35] that includes a steering committee comprising 8 stakeholders (researchers, workers, managers, and rehabilitation professionals). Such a design engages researchers and participants in a reflective process as, together, they seek solutions to an issue. It enables the production of useful and accurate knowledge that corresponds to stakeholders’ realities [36]. Therefore, this study will focus on aging workers who have returned to work after an absence during the past 2 years and experienced telework as an accommodation modality. Managers and rehabilitation professionals will also participate in the study to provide other perspectives. The research involves the following three phases: (1) exploration; (2) design, validation, and drafting; and (3) testing and evaluation. Figure 1 shows the phases of the research leading to the development of the reflective guide.

### Procedure, Participants, and Analysis

#### Phase 1

This phase will address objective 1 in 3 steps.

##### Overview

Recruiting all participants in this study will occur through social media announcements and contacts with clinical settings and organizations within the research team member networks. We will use a purposive sampling method to recruit participants and a maximum variation sampling strategy to select them [37]. Recruiters will ensure diversity in terms of gender, age, and employment sector.

##### Step A

A 60-minute individual web-based interview with 10 aging workers, respectively, will address various themes (eg, work practices, life role balance, feeling inclusion and satisfaction at work, and perceived health status) using a pretested interview guide. Eligibility to participate will depend on candidates being at least 55 years of age and having experienced telework as an accommodation modality upon returning to work after an absence during the previous 2 years.

##### Step B

A 60-minute individual web-based interview will occur with each of the 10 managers working with aging workers, addressing themes similar to those in step A with an emphasis on management practices and using an equivalent pretested interview guide. Managers will have accommodated at least one aging worker with telework after an absence during the previous 2 years.

##### Step C

A 60-minute individual web-based interview will occur with 10 rehabilitation professionals, respectively. Recruits will be working in occupational rehabilitation (ie, occupational therapists, physiotherapists, and psychologists). Each interview will address themes similar to those in step A, emphasizing rehabilitation practices and using an equivalent pretested interview guide. Eligible rehabilitation professionals will have accompanied at least one aging worker returning to work through telework after an absence during the past 2 years.

A member of the research team will conduct all interviews, digitally audio-recording each. Based on the available literature [38,39], a total of 30 participants should enable reaching content saturation. A questionnaire will collect sociodemographic data (eg, age, gender, marital status, employment experience, and health status) for each participant.

##### Analysis

Following the transcription of interviews, 2 research team members will analyze the data using an inductive posture and a 5-step thematic-analysis strategy [40]: (1) repeated readings of the data corpus to enable researchers to develop a sense of immersion; (2) initial coding with descriptive codes assigned to the units of significance found in the corpus; (3) units of meaning transformed into meaningful expressions of participants’ perspectives; (4) synthesis of expressions allowing the organization of the data in a general structure; (5) back and forth between the raw data and the general structure, allowing clarification and interpretation of the data while respecting participants’ perspectives. The 2 coders will first analyze each transcript individually, then compare their structure of codes and generate a new, improved version. This process prevents the analysis from reflecting a single person’s perspective and ensures interjudge agreement. The research team may produce several versions of the structure until the team agrees on a structure that most accurately reflects the data. QDA Miner software will support the analysis process.

The researchers expect that the results obtained in this phase will enable generating various elements, such as questions to ask, factors to consider, levers, good practices, and obstacles to avoid, to promote the accommodation, inclusion, and health of aging workers returning to work after an absence.

#### Phase 2

This phase will address objective 2 in 3 steps.

##### Step D

Identified Phase 1 elements will support designing the logic model of a reflective telework application guide for aging workers and their managers. The logic model will present the goals, supporting theories, components, sections, and expected outcomes of its use [41]. Some authors have suggested the added value of creating a logic model in the process of developing a new tool, especially because this preliminary step facilitates communication between the actors involved and promotes their common understanding of the tool to be developed [41,42]. The steering committee will develop this logic model using a co-construction approach during a working day.

##### Step E

Validation of the logic model will involve a 120-minute web-based group using the “technique for research of information by animation of a group of experts (TRIAGE)” [43,44], namely, a group of 8 participants in Phase 1. The TRIAGE technique allows structuring discussions between participants to obtain a consensus on specific ideas while encouraging the emergence of new ones [44]. Two research team members will facilitate the group. Various indicators of validity related to logic models (ie, relevance and clarity) will be documented [41] with a pretested guide. Data analysis related to the validation of the logic model will occur during the session (in situ), consistent with the TRIAGE technique [44].

##### Step F

Based on this validation of the logic model, the steering committee will create the first version of the complete guide during a second working day.

#### Phase 3

This phase will address objective 3 in 3 steps.

##### Step G

This phase aims to test and evaluate the guide with respect to the accommodation, inclusion, and health of aging workers. It will determine whether users of the guide consider it appropriate, particularly in terms of acceptability [45] and satisfaction. We will invite 6 teleworker-manager dyads to familiarize themselves with the guide and use it in their daily work for a month.

##### Step H

Following this trial period, the perceptions of the dyads will be collected in a semistructured interview to document various indicators of acceptability (eg, affective attitude, perceived effectiveness, and opportunity costs [45]), and satisfaction regarding accommodation, inclusion, and health. Qualitative open-ended questions will support this purpose (“Which barriers to the use of the guide have been faced?” “How much has the guide facilitated a sense of inclusion at work?”) along with quantitative closed-ended ordinal scale questions (“What is your level of satisfaction regarding the use of the guide?”). A thematic analysis strategy [40] will support analyzing qualitative data, and descriptive statistical analysis (eg, frequency, means, and SDs) will enable quantitative data analysis [46].

##### Step I

Finally, applying the same measures to the same participants in step G will occur at 6 and 12 months to document the sustainability of the guide’s use and its effects on the accommodation, inclusion, and healthy work participation of aging workers.

### Ethics Approval

This study obtained ethical approval from the Ethics Committee of the Centre intégré universitaire en santé et services sociaux de la Capitale-Nationale (project 2023-2805) and the Human Research Ethics Committee of the Université du Québec à Trois-Rivières (project CER-23-298-10.01)

## Results

Data collection began in spring 2023 and initial results are expected in fall 2023. This study aims to produce a concrete tool, namely, a reflective telework application guide for short-term use by rehabilitation professionals, managers, and aging workers, to improve the implementation of telework as an accommodation modality to support the inclusion and health of aging people in their return to work after an absence. The guide will also serve to influence policy makers regarding telework. Since rehabilitation professionals and managers are often poorly equipped to respond to such a unique population, the results should propose guidelines for using telework to return to work.

To share the results of the project and increase its sustainability potential, the steering committee will organize several dissemination activities, either during the project or at its end. The researchers will frequently post the study’s progress on social media and in a newsletter. We will record a podcast with a researcher, a worker, a manager, and a rehabilitation professional to publicize the guide and its potential utilization. We will invite various stakeholders in the world of work (ie, unions, insurers, and employer and worker associations) to listen to this podcast. Also, we will submit the results for publication in scientific journals and presentation at international scientific conferences. To ensure rigor in the dissemination of results, we will follow the Standards for Reporting Qualitative Research (SRQR) [47]. Finally, we will promote the use of the guide to government authorities.

## Discussion

### Overview

This study will create a reflective telework application guide through a rigorous, multistep research process involving various work-related stakeholders. This study constitutes a first of its kind and is highly relevant, especially in the context of the labor shortage and the recent major changes in the world of work. To our knowledge, there are no tools, guidelines, or “best practices” for rehabilitation professionals, managers, or workers to use to ensure a healthy application of telework, specifically for the aging population. This project is particularly innovative on several levels. On a *practical level*, the guide represents a concrete tool that rehabilitation professionals, managers, and aging workers can use upon returning to work after an absence. This study’s *research* contribution is to pave the way for other projects aimed at developing innovative and effective solutions in promoting the accommodation, inclusion, and health of aging workers after an absence. Finally, on a *societal level*, the study results will help to explain the influence of telework on the health and inclusion of aging workers and encourage people to use the guide to promote healthy participation in telework.

### Conclusion

This paper presents a research protocol to develop a reflective telework application guide with its basis in scientific and experiential knowledge. The results of this study will lead to implications at practical, scientific, and societal levels. Moreover, the results of this study should pave the way for further studies and interventions to ensure aging workers’ healthy work participation.

## Appendix

Multimedia Appendix 1 Peer-reviewer report from Réseau Québécois de Recherche sur le Vieillissement (Quebec Network for Research on Aging, Quebec, Canada).

## Abbreviation

TRIAGE: technique for research of information by animation of a group of experts

## Data Availability

The data sets generated and analyzed during this study are available from the corresponding author on reasonable request.

## References

[1] Government of Canada (2017). Census in brief: working seniors in Canada. Statistics Canada.

[2] Fields A, Uppal S, LaRochelle-Côté S (2017). L'incidence du vieillissement de la population sur les taux d'activité du marché du travail. Statistique Canada.

[3] Toossi M, Torpey E (2017). Older workers: labor force trends and career options. Career Outlook.

[4] U.S. Department of Labor The economics daily: number of people 75 and older in the labor force is expected to grow 96.5 percent by 2030. Bureau of Labor Statistics.

[5] European Union (2022). Taux d'emploi des personnes agées, tranche d'âge 55-64 ans. Eurostat.

[6] Twenge JM, Campbell SM, Hoffman BJ, Lance CE (2010). Generational differences in work values: leisure and extrinsic values increasing, social and intrinsic values decreasing. J Manage.

[7] Hansen T, Slagsvold B (2020). An "Army of volunteers"? Engagement, motivation, and barriers to volunteering among the baby boomers. J Gerontol Soc Work.

[8] Benson J, Brown M (2011). Generations at work: are there differences and do they matter?. Int J Hum Resour Manag.

[9] McCann RM, Keaton SA (2013). A cross cultural investigation of age stereotypes and communication perceptions of older and younger workers in the USA and Thailand. Educ Gerontol.

[10] Lecours A, Robitaille R (2020). Comment le travail après la retraite influence-t-il la santé des travailleurs vieillissants? Un examen de la portée. Recueil annuel belge d'ergothérapie.

[11] Busque MA, Duguay P (2017). Lésions avec atteinte permanente à l'intégrité physique ou psychique: analyse du risque au Québec. Institut de recherche Robert-Sauvé en santé et sécurité du travail.

[12] Van Eerd D, Smith P, Vu U (2019). Implications of an aging workforce for work injury, recovery, returning to work and remaining at work. J Ontario Occup Health Nurses Assoc.

[13] Fraade-Blanar LA, Sears JM, Chan KCG, Thompson HJ, Crane PK, Ebel BE (2017). Relating older workers' injuries to the mismatch between physical ability and job demands. J Occup Environ Med.

[14] Gopinath B, McMahon CM, Burlutsky G, Mitchell P (2016). Hearing and vision impairment and the 5-year incidence of falls in older adults. Age Ageing.

[15] Boucher A, Duguay P, Busque M-A (2019). Analyse des différences de durées d’indemnisation selon le sexe et le groupe d'âge. Institut de recherche Robert-Sauvé en santé et sécurité du travail.

[16] Neary J, Katikireddi SV, Brown J, Macdonald EB, Thomson H (2019). Role of age and health in perceptions of returning to work: a qualitative study. BMC Public Health.

[17] (2014). Portrait des lésions professionnelles chez les travailleurs de 55 ans et plus, 2002-2011. l’Équipe des études et analyses du Centre de la statistique et de l’information de gestion de la Direction de la comptabilité et de la gestion de l’information.

[18] Evans DM, Conte K, Gilroy M, Marvin T, Theysohn H, Fisher G (2008). Occupational therapy: meeting the needs of older adult workers?. Work.

[19] Lecours A, Bédard-Mercier R (2023). L'expérience de retour au travail des personnes vieillissantes ayant subi une atteinte à la santé: un examen de la portée. Can J Aging.

[20] Jauvin N, Stock S, Laforest J, Roberge M-C, Melançon A (2020). Le télétravail en contexte de pandémie: mesures de prévention de la COVID-19 en milieu de travail. Institut national de santé publique du Québec.

[21] Zossou C (2021). Partage des tâches domestiques: faire équipe pendant la pandémie de COVID-19. Statistique Canada.

[22] (2021). La COVID-19 au Canada: le point sur les répercussions sociales et économiques après un an. Statistique Canada.

[23] Ipsen C, van Veldhoven M, Kirchner K, Hansen JP (2021). Six key advantages and disadvantages of working from home in europe during COVID-19. Int J Environ Res Public Health.

[24] Magnavita N, Tripepi G, Chiorri C (2021). Telecommuting, off-time work, and intrusive leadership in workers' well-being. Int J Environ Res Public Health.

[25] Yang Y, Li W, Zhang Q, Zhang L, Cheung T, Xiang Y-T (2020). Mental health services for older adults in China during the COVID-19 outbreak. Lancet Psychiatry.

[26] Heisey AG (2012). Teleworking: work/life balance of online instructors with disabilities: a phenomenological study. Capella University.

[27] Tang J (2021). Understanding the telework experience of people with disabilities. Proc ACM Hum-Comput Interact.

[28] Moon NW, Linden MA, Bricout JC, Baker PMA (2014). Telework rationale and implementation for people with disabilities: considerations for employer policymaking. Work.

[29] Till M, Leonard T, Yeung S, Nicholls G (2015). Profil des expériences sur le marché du travail: adultes canadiens de 15 ans et plus ayant une incapacité, 2012. Statistique Canada.

[30] Linden M (2014). Telework research and practice: impacts on people with disabilities. Work.

[31] Linden M, Milchus K (2014). Teleworkers with disabilities: characteristics and accommodation use. Work.

[32] Schur LA, Ameri M, Kruse D (2020). Telework after COVID: a "Silver Lining" for workers with disabilities?. J Occup Rehabil.

[33] Anderson J, Bricout JC, West MD (2001). Telecommuting: meeting the needs of businesses and employees with disabilities. J Vocat Rehabil.

[34] Hamouche S, Parent-Lamarche A (2022). Teleworkers' job performance: a study examining the role of age as an important diversity component of companies' workforce. J Organ Eff People Perform.

[35] Contandriopoulos AP, Bélanger L, Nguyen H (1990). Savoir préparer une recherche: la définir, la structurer, la financer.

[36] Reason P, Bradbury H (2001). Participative inquiry and practice. The SAGE Handbook of Action Research.

[37] Patton MQ (2002). Qualitative Research & Evaluation Methods, 3rd ed.

[38] Hennink MM, Kaiser BN, Marconi VC (2017). Code saturation versus meaning saturation: how many interviews are enough?. Qual Health Res.

[39] Guest G, Bunce A, Johnson L (2016). How many interviews are enough?. Field Methods.

[40] Paillé P, Mucchielli A (2021). L'analyse qualitative en sciences humaines et sociales, 5 ed.

[41] Porteous NL, Ridde V, Dagenais C (2012). La construction du modèle logique d'un programme. Approches et pratiques en évaluation de programme.

[42] McLaughlin JA, Jordan GB, Newcomer KE, Hatry HP, Wholey JS (2015). Using logic models. Handbook of Practical Program Evaluation.

[43] Gervais M, Pépin G (2002). TRIAGE: a new group technique gaining recognition in evaluation. Eval J Australas.

[44] Albert V, Durand MJ, Pépin G (2020). TRIAGE: une technique structurée sollicitant l'opinion d'experts en vue d'atteindre un consensus. Méthodes qualitatives, quantitatives et mixtes dans la recherche en sciences humaines, sociales et de la santé, 2 ed.

[45] Sekhon M, Cartwright M, Francis J (2017). Acceptability of healthcare interventions: an overview of reviews and development of a theoretical framework. BMC Health Serv Res.

[46] Field AP (2013). Discovering Statistics Using IBM SPSS Statistics: and Sex and Drugs and Rock 'n' Roll.

[47] O’Brien BC, Harris IB, Beckman TJ, Reed DA, Cook DA (2014). Standards for reporting qualitative research. Academic Medicine.

